# Family burden of schizophrenic patients and the welfare system; the case of Cyprus

**DOI:** 10.1186/1752-4458-7-13

**Published:** 2013-05-02

**Authors:** Christos Panayiotopoulos, Andreas Pavlakis, Menelaos Apostolou

**Affiliations:** 1 Department of Social Sciences, University of Nicosia, 46 Makedonitissa Avenue, Nicosia 1700, Cyprus; 2 Department of Health Management, Open Uiversity of Cyprus, 5 Ayios Antonios, Strovolos, Nicosia, 2002, Cyprus

## Abstract

**Background:**

The shift from asylum to community care for mental health patients has burdened the providers of primary health care and, more than all, families. As a result, numerous studies [*Soc Psychiatry Psychiatr Epidemiol* 31:345–348, 1995, *J Health Socisl Behav* 36:138–150, 1995] have focused on the burden of care experienced by family members living with individuals with severe mental disorders. This kind of provision, also extols a significant cost to the society at large in terms of significant direct and indirect costs. A cost that may be even higher in times of severe socio-economic crisis.

**Methodology:**

This study, firstly, aims to examine the burden that the family members experience by caring for individuals with schizophrenia and the identification of the parameters, in a micro and macro level, that affect family burden. Secondly, this study aims to investigate whether the welfare state will be fit to help vulnerable groups as the one studied, especially during economic crisis periods when austerity measures are being implemented into welfare systems. For data collection purposes this study employed the Involvement Evaluation Questionnaire [*Schizophr Bull* 1998, 24(4):609–618]. The sample consisted of caregivers either living in rural or urban areas of the district of Nicosia, the capital of the Republic of Cyprus. These people were attending regular meetings with their allocated Community Psychiatric Nurses (CPN) in Community Mental Health Centres (CMHC).

**Results:**

Analysis of covariance (ANCOVA) was applied with the tension, the supervision, the worry, and the encouragement entering as dependent factors. In each case, participant’s age, gender, marital status, income, number of people living in the same house with the participant, degree of relationship between the caregiver and the person suffering from severe mental disorder, the age of the relative, and the gender of the relative, were entered as independent factors. Four ANCOVAs were performed, one for each dimension of the family burden. The results from this analysis produced only one significant main effect of the gender of the relative on supervision [F(1,118) = 4.40, p = .011, etap2 = .053] with male relatives suffering from schizophrenia requiring higher supervision than female ones as their relative caregivers responses indicate.

**Conclusions:**

Consequently, families under great stress due to the reasons derived from the weaknesses of the welfare system described throughout this paper would give up and reject the mentally ill individuals who would become outcasts socially. Therefore, health systems need to aim to the development of psychosocial provisions for both family caregivers and patients as to decrease the family burden rates and increase the possibility of smooth transition to the society.

## Introduction

During recent years the treatment of mental health patients has improved vastly by the provision of community care and rehabilitation. Despite this debate amongst institutionalization and de-institutionalization progressively, the responsibility of care moved onto families who act as the frontline caregivers. Hence, families are now confronted with multifaceted problems such as fear and anxiety in relation to the patient’s symptoms and the cost of treatment at home [1]. The trend towards community care offered to mental patients has burdened the providers of primary health care and, more than all families. As a result, numerous studies [2,3] have focused on the burden of care experienced by family members living with individuals with severe mental disorders. This kind of provision, also extols a significant cost to the society at large in terms of significant direct and indirect costs, such as frequent hospitalizations and the need for long-term psychosocial and economic support, as well as life-time lost productivity. This cost that can possibly be higher in times of severe socio-economic crisis. In particular, Phua [4], 2011 claims when economic conditions worsen, we should expect increased poverty levels, foreclosures on houses, homelessness and related housing problems. More specifically, the economic crisis that started in 2007 has continued to pose major challenges especially in the European Region. It has led to significant declines in economic activity, a rise in unemployment, depressed housing markets and an increasing number of people living in poverty [5], p.6. The rise in national debt is forcing governments to implement severe cuts in public spending. Significant risks remain in the world economy, and many countries are facing an era of austerity in health and welfare services. Those opposing the community mental health movement would argue that the shift towards community mental health has lead on the one hand to the reduction in psychiatric inpatient beds since the 1950s, but would strongly argue that this reduction has been more or less equaled by an increase in the provision in the private sector for this group, the so called ‘virtual asylum’ [6]. In line with the opposing group to community care is the fact that, many health and social service localities find it impossible to provide sufficient residential and nursing home places for those leaving hospital and outsource to facilities away from the local area. Concerns also rise onthe quality and continuity of care for people placed in these ‘out of area treatments’ (OATs) [7]. There is also concern that those commissioning these services have inadequate information about the clients they place there, the facilities provided and lack of systems for review of placements. In the case of UK, for example, it is claimed that the cost of the “virtual asylum” provisions to the National Health System (NHS) alone has been estimated at £222 million per year [8]. This process of ‘re-institutionalistion’ appears to be taking place elsewhere in Europe too [9].

In light of the above challenges, this study’s aim is twofold. Firstly, it aims to examine the burden that the family members experience by caring for individuals with schizophrenia and the identification of the parameters, in a micro and macro level, that affect family burden. Secondly, following research evidence [4] indicating the significant relationship between unemployment and poor health at both the level of the population and the individual, this study aims to investigate whether the welfare state will be fit to help vulnerable groups as the one studied during economic crisis periods with austerity measures being implemented onto welfare systems. Therefore, the study presented in this paper addresses the following research questions:

1. What are those variables that affect the family burden of caregivers of schizophrenic patients?

2. Is there a possible relationship between family burden and the provisions of the Cypriot welfare state to mentally ill patients?

### Setting the scene

The burden of care imposed on a family may be negatively linked to the overall level of family function [10]. With regard to the attitudes of relatives of patients with schizophrenia it should be noted that the social stigma attached to mental disorders contributes to feelings of frustration and anger. Families are forced to acknowledge the stark reality of having a member with schizophrenia and to mourn the loss of unfulfilled expectations. Moreover, as a result of the chronic stress associated with the task of caring [11], it is common for families to have emotional responses such as anxiety, fear, guilt, stigma, frustration, anger, and sadness to say the least.

Experiencing *burden of care* is a complex construct that challenges simple definition, and is frequently criticized for being broad and generally negative [12]. Frequently, *burden of care* is more defined by its impacts and consequences on caregivers [13]. In addition to the emotional, psychological, physical and economic impact, the concept of *burden of care* involves subtle, but distressing notions such as shame, embarrassment, feelings of guilt and self-blame. The early conceptualization of *burden of care* into two distinct components (objective and subjective) has guided research efforts until the present time. *Objective burden* of care is meant to indicate its effects on the household such as taking care of daily tasks, whereas *subjective burden* indicates the extent to which the caregivers perceive the burden of care [14]. In particular in schizophrenia which as a chronic psychiatric disorder poses numerous challenges in its management and consequences. Indicative of this situation is a claim from a study conducted by Marsh et al., [15], 1996 where a family member said “that this terrible disease stigmatises everything- the family cannot escape from this trap”. Another study with a Latin American sample [11] evidenced that relatives of patients with schizophrenia showed high levels of burden as a result of their care task. Moreover, the same study provided evidence that the financial problems, the restriction of spare time, and the patient’s future, were of considerable concern to caregivers. Some of these concerns can be attributed as objective and/or subjective burdens.

It is therefore imperative to acknowledge that family burden is about to increase as an economic crisis affects the factors determining mental health. Especially as burden of family caregivers leads to negative consequences not only for themselves, but also for patients, other family members, and health care system [16]. More specifically, protective factors (i.e. social capital and welfare protection, healthy workplace and living) [5] are weakened and risk factors (i.e. poverty, deprivation, high debt, job insecurity, unemployment) strengthened. In line with WHO recent report on the impact of economic crisis on mental health is Matsaganis’ (2011) claim that the current financial crisis and the measures to counter it are affecting the welfare state profoundly, and in various aspects [17]. This is so, as the fiscal crisis is depriving the welfare state of precious resources. In the poorer countries of the developing world, economic crisis may force governments to cut back drastically on health spending, forcing poor patients to seek care from NGOs, delay care seeking, self-medicate or even go without care completely [4]. A characteristic example of this situation is the case of the National Health Service (NHS) in UK which is funded by general tax revenue; the prolonged economic crisis would generate pressure (as well as the political opportunity) to cut spending on social services such as health in order to reduce the size of the budget deficit in spite of rising demand for such services. More related, to this study and especially to its second aim, is the case of Greece that has been lead to cut back in fiscal resources as far as mental health operational system concern.

Despite the lack of a National Health System in Cyprus, where this study is located, the state has been proved rather generous in providing welfare benefits that perhaps alleviate the burden to both the patient and the caregiver in many aspects across the health sector [18]. This is characteristically pointed out in Christofides [19], 2011 brief report about unemployment insurance and social welfare in Cyprus and Amitsis’ (2012) article; both provide ample evidence on a general comprehensive scheme of guaranteed minimum income (Public Relief Scheme), which provides means – tested benefits and personal social services. However the socio-economic status of the Republic of Cyprus is about to change drastically as recently it has been asked to apply austerity measures and to cut back on a series of welfare benefits ^a^such as single parents’ benefits, public relief scheme, unemployment benefits. This is anticipated to have an impact on the caregivers of mentally ill people as the latter fall under the vulnerable group category and in addition they will have to pay for a part of their treatment received by public medical services including psychiatric services.

Embarking on this endeavor this study was confided in exploring only one group of family caregivers. In particular research was conducted with family caregivers of schizophrenic patients because research indicates that this mental disorder has a considerable impact on patients and their families [20,21].

## Methodology

The primary aim of this study is the investigation of the level of *burden of care* as experienced by families of schizophrenic patients. Especially, this study investigated the impact on caregivers and the parameters that affect and describe the burden families experience. Secondary aims of this particular study were the following:

• An in depth analysis of the emotional state of caregivers

• Quality of caring that caregivers provide to their relative/s mental health patients

• Concern about the feeling of security as far as the mental well-being of the patient

• Design and implementation of interventions for family members aiming to alleviate or decrease the burden.

### Compliance with bioethics

On the 20^th^ of August 2009 an approval was given by the scientific committee of the Cypriot Ministry of Health and the director of Mental Health Services as far as the ethics of the study. The participation of individuals in the study was determined after each subject was informed for all the details concerning the study and consented to it, according to the WHO (2000)^b^ rules for bioethical research and deontology for the protection of participants.

### Participants (sample)

Participants in this study were relatives of individuals who have been diagnosed with schizophrenia, and who were responsible for the caring at home. Participants were either living in rural or urban areas of the district of Nicosia, the capital of the Republic of Cyprus. These people were attending regular meetings with their allocated Community Psychiatric Nurses (CPN) in Community Mental Health Centres (CMHC). The overall population of patients who live in the community and have been diagnosed with schizophrenia is 165 as shown in Table 1. Therefore, the population (family caregivers) was 165, in total. As the only inclusion criterion was the psychiatric diagnosis and the cohabitation with the patient out of the total number of possible participants, only 127 consented in participating in the study. The other 20 were used in the pilot phase and the remaining 18 of the research sample refused to participate due to their age and inexperience from scientific studies.

**Table 1:** Sampling

**Number of mental health patients who live in the community and are seen by CPN in the area of Nicosia**	**Number of schizophrenic patients who are seen by CPN in the area of Nicosia**	**Pilot Study**	**Participants in the study who gave their consent**	**Participants who refused to participate in the study**
**940**	**165**	**20**	**127**	**18**

### Research tool

For data collection purposes this study used the Involvement Evaluation Questionnaire (EUROPEAN VERSION/ IEQ – EU) [22]. This instrument has been used in similar studies in several European countries (i.e., Holland, Denmark, England, Italy, Spain) and was found to be reliable and valid [23]. The questionnaire requires that the relative or the caregiver is in contact with the individual suffering from a severe mental illness at least an hour every week for the last four weeks.

The questionnaire was divided in three sections. In the first section, demographic information was collected (age, gender, marital status, income, number of people living in the same house with the participant, degree of relationship between the participant and the person suffering from severe mental disorder, the age of the relative, the gender of the relative). In the second section, participants had to indicate their agreement or disagreement with a number of statements using a five-point Likert scale (1-never, 2-sometimes, 3-frequently, 4- very frequently, 5- almost always) in relation to worry about the mental state of the patient, encouragement of the latter, problems in communication with the mentally ill, etc. In the last section, participants were asked about the financial expenses with respect to their relative’s care. The answers were dichotomous (yes/no) with the exception of the variable which measures how much money in total the participants had to spend in the previous month to support their relative, which had five levels (less than 50 euro, 50–80 euro, 80–200 euro, 200–400 euro, more than 400 euro).

This instrument measures four aspects of family burden namely, tension, worry, supervision, encouragement, and financial costs. In more detail, tension refers to the harmony of the relationships between the participant and the relative with the serious mental health problem. The worry dimension refers to the anxiety that the participants feel for the safety, the health and the medical care their relatives who suffer from serious mental disorder receive. Supervision refers to the responsibilities that participants have for their mentally ill relatives, such as making sure that their relatives take their medication or sleep properly. The encouragement dimension refers to the encouragement that the caregiver gives to the persons with the disorder so as to enable them to look after themselves, eat properly etc. Finally, the financial burden dimension explores the financial costs that the care of a relative with a serious mental disorder incurs.

Despite the fact that this questionnaire has been validated in Greece [24] we also pilot tested it in order to adapt it in the local Cypriot context should this arise as necessary. Pilot study results indicated no difference to the one conducted in Greece and they did not reveal any problems as far as comprehension or suitability of the questionnaire items.

### Research process

The questionnaire was completed during personal interviews with the study participants (family relatives) and the psychiatric nurse who keeps a close contact with the relatives. The interviews lasted approximately an hour and took place at the each participant’s home. All participants had to sign a consent form before the beginning of the interview. Prior to this meeting all potential participants had been informed through telephone contact about the study. Community Psychiatric Nurses informed all participants about the purposes of the study and the anonymity and the confidentiality of their responses.

### Data analysis

#### Results

This section is organized to present the basic results as these rose following data analysis, using the SPSS. In particular the first section of the results aims to describe the sample through presenting some basic demographic data. The next results section is closely associated with the four aspects of family burden namely, tension, worry, supervision, encouragement, and financial costs.

## Results

### Demographics

Overall, in this study 127 Greek-Cypriot caregivers (97 women and 30 men) took part; participants were identified as family caregivers with one family member who suffered from schizophrenia. Of the participants 97 were female (46.8%) and 30 were male (53.2%). The mean age of female participants was 58.8 (*SD* = 15.33), and the mean age of male participants was 60.2 years (*SD* = 12.6). Moreover, 68.5% of the participants were married, 18.9% were widowed, 6.3% were single, and 6.3% were divorced. In addition, 45.6% held a primary school degree, 37.6% held a high school degree, and 16.8% held a university degree. Also, the mean age of the male relatives was 46.12 (*SD* = 15.31) and the mean age of female relatives was 47.35 (*SD* = 13.96). In 78% of the cases participants indicated that their relative was staying with them. Finally, in 50% of the cases participants reported that their relative who suffered from schizophrenia was their father or mother, in 22% of the cases was their spouse or partner, in 16.7% was their offspring and in 11.1% other sibling.

### Family burden’s aspects analysis

In order to estimate the effects of the severe mental disorder, in this case schizophrenia, to the family unit we calculated the mean score of participants’ responses for the tension, the supervision, the worry and the encouragement dimensions of the family burden. For the financial burden we estimated the relative frequencies of participants’ responses for each question related to this dimension.

In relation to the dimension *tension*, the mean score was 2.11 (*SD* = .72). This result indicates a relatively low tension in the relationship between participants and their relatives who suffer from schizophrenia. In particular, the majority (54.55%) of the sample replied that they worry quite often about the security of their relative, while 63.7% of the research sample is concerned about the economic situation of the family members in the event caregivers will no longer be able to offer their support. Results concerning the dimension *supervision* a mean score 1.62 (*SD* = .55) was reported indicating that the relative’s mental disorder imposes low supervision demands to the participants.

With respect to the dimension *worry* as found in the scale, the mean score was 3.13 (*SD* = 1.03) indicating that participants have an above average level of worry for their relatives’ welfare. Moreover, in relation to the dimension *encouragement* the mean score was 3.07 (*SD* = .96) indicating that participants have frequently to devote energy in encouraging their mentally ill relatives to look after themselves.

Moving on to the financial burden dimension, when participants were asked to indicate the extra money they spent for the needs of their relative, 33.6% reported that they spent less than 50 euros, 29.3% between 50 and 80 euros, 22.4% between 80 and 200 euros, 8.6% above 400 euros, and 6% between 200 and 400 euros. Thus, for 63% of the participants in our sample the financial burden is small, and only for approximately 15% of them is relatively large. This financial burden can be broken down as follows: 20.5% of the participants indicated that they had to spend extra money to help a relative with his/her employment obligations, 11% to cover the costs of damages caused by their relative, 15.7% for various non-trivial expenses, 14.2% for traveling expenses, 31.5% for medication expenses, and 12.6% for paying their relatives’ debts.

### Family burden contingencies

In order to examine further whether the various aspects of family burden were contingent upon the demographic factors recorded in this study further analyses were performed. In this respect, analysis of covariance (ANCOVA) was applied with the dimension *tension*, *supervision*, *worry*, *and encouragement* entering as dependent factors. In each case, participant’s age, gender, marital status, income, number of people living in the same house with the participant, degree of relationship between the participant and the person suffering from severe mental disorder, the age of the relative, and the gender of the relative, were entered as independent factors. Four ANCOVAs were performed, one for each dimension of the family burden (i.e., the tension, the worry, the supervision, the encouragement).

To estimate possible contingencies on the financial burden, the extra money spent for the relative with severe mental disorder was enter as the dependent variable. As this variable is measured in an ordinal rather than in an interval scale, ordinal regression was applied with the same set of independent factors. In both cases (ANCOVA and ordinal regression), backward elimination was employed in order to reach an optimal model.

The results (Table 2 from this analysis produced only one significant result. In particular, there was a significant main effect of the gender of the relative on supervision [*F*(1,118) = 4.40, *p* = .011, *ηp*^*2*^ = .053] with male relatives suffering from schizophrenia requiring higher supervision than female ones as their relative caregivers responses indicate.

**Table 2:** Family burden contingencies

	***Tension***		***Supervision***		***Worry***		***Encouragement***		***Financial burden***	
*Predictors*	F	P	F	P	F	P	F	P	Wald	P
Age	.367	.548	.323	.573	1.78	.196	.200	.657	1.75	.186
Sex	1.96	.168	.257	.856	1.42	.250	.552	.649	.928	.335
Marital Status	.058	.981	.573	.749	.775	.593	.445	.844	.035	.852
Income	.870	.524	.763	.387	.042	.838	.684	.413	1.93	.159
People living in the same house	.457	.714	.820	.489	1.92	.138	.186	.906	1.80	.172
Degree of relationship	.770	.385	.077	.783	.783	.612	.060	.808	1.15	.282
Age of the relative	1.30	.203	1.41	.198	.621	.434	.633	.431	.283	.595
Sex of the relative	.070	.792	4.40	.011	.723	.410	.655	.401	3.59	.058

## Discussion

Despite the fact that a high proportion of family members providing care to persons with schizophrenia experience high rates of burden [25] equivalent to that of caregivers of persons with other neurological(e.g., Alzheimer’s disease, mental retardation) and physical (e.g., diabetes, cancer) disorders [26,27] the results from our analysis indicate that a severe mental disorder such as schizophrenia has a relatively low burden (mean = 2.49, st.d = 0.837) for the Cypriot family. In particular, the only aspects of the family burden that seem to agree with other studies are *the worry and the encouragement* dimensions. An expected result if we take in consideration that Cypriot families tend to keep away from the public domain the existence of a member with a disability or mental illness [28].This view is also supported by other research evidence [29]. As a result of not sharing their difficulty with other supportive systems (i.e. community mental health services, wider society, neighborhood) they retain their collectivist culture and, tend to seek help only from close friends and family members when suffering from mental illness and may have minimal contact with psychiatric services [30,31].Thus, it is more likely that they will try to conceal it and only contact psychiatric services if the symptoms are extremely severe or when they are not capable of taking care their relatives [31-33]. Another finding that makes this result justifiable is that the majority of the caregivers are above 50’s. According to Fujino and Okamura (2009) the older a caregiver becomes, they are more worried about who will take care of their ill family member in the future [34].

Those two aspects of family burden might not be the most burdensome though in comparison with those of *tension*, *supervision* and *financial cost*. However, their mean scores indicate that they are not influenced considerably by the nature of schizophrenia; even so the fact that burden is experienced is in line with other studies which have indicated that family members of schizophrenic patients experience burden on a practical, financial and emotional level (Expressed Emotion) and the extent of the burden is closely linked to the amount of symptomatic behaviour of the patient [35]. This can be argued to be a rather surprising result that demands explanation in various aspects. With respect to *the tension and the supervision* dimensions, someone could argue that modern medication may reduce substantially the symptoms of schizophrenia, making patients more able to lead a normal life. In turn, this predicts less tension between them and their relatives and less need for supervision. However, according to the bio-psychosocial model (ref), medication has proved to be the one side of the treatment as the psychosocial part is equally important to the integration of psychiatric patients into the community. However, Cypriot caregivers reported not having the knowledge and skills necessary to take on the responsibilities of caregiving for these relatives [36,37]. It could then be argued that the explanation behind this rather paradox result lies again on the family culture that exists in the local context; the family orientated context of Cyprus has similar traits with …..These are the close bonds amongst family members, and the fact that matters are being dealt with within the family system even though external help could be needed [38]. In Southern European countries it is taken for granted in many cases that it is up to the household to provide for the welfare (housing, health) of their members and therefore no emphasis is placed on specialized welfare family policies such as psycho-education for caregivers and family counseling (ref). In contrast with the above in other countries such as the USA and the UK, individualism and self-efficiency in their family policies are promoted. According to Lewis (1995) in the US, for example, little attempt is made to subsidize women to drop out of the work force, but there is also little attempt to subsidize family caregiving for working mothers. Social supports for working mothers and their children are primarily reserved for only the most impoverished families. Italy, Greece and other Southern European countries on the other hand, encourage women to drop out of the work force for long periods of time by offering lengthy paid leave, which get much higher fiscal and social priority than investments in child care. Inthe “liberal”welfare state (i.e. USA) the state caters for the very low income groups In the Southern European/Mediterranean (SE/M) welfare state [39] , social benefits are provided to a large number of recipients (single-parent families, asylum seekers, disabled, child care benefits). Cyprus belongs to the SE/M welfare state and based on the results of this study we can see the need for Cyprus to restructure and reshape its welfare state into a welfare regime that will have to secure both the fiscal and the social survival of vulnerable groups and their relative [40].

The fifth dimension of the burden investigated in this study was the *financial burden* taken by the caregivers. Andren and Elmstahl (41, 2006) conducted a study in Sweden to examine the relationship between income, subjective health and caregivers’ burden in people with dementia. Finding showed that low income was associated with a higher degree of burden on the caregivers as by providing care for ill member, they also had to solve financial problem and find out source of money. Our results seem to counter act Andren and Elmstahl results [41], since in our study caregivers who reside with schizophrenic patients do encounter more financial hardships. A 14,6% of the sample said that spends over 400 euros to cover medical and other expenses due to the illness of their family member. Looking at this result we reach the conclusion that the low financial burden might be due to a wealthy welfare benefits’ system. In particular, social benefits / services for needy persons in Cyprus form part of a broader regulatory framework concerning poor people and other socially excluded persons [42]. The personal scope of the Public Relief Scheme is quite vast, since according to article 3 of Law 8/1991 “Public benefits according to the provisions of this Law are allocated to any person, who is a permanent resident of Cyprus and whose income or other economic resources are not adequate for his/her basic and special needs, after submitting an application to the Director of Social Welfare Services Department or to any of his legitimate representatives”. In particular, those diagnosed with a severe mental disorder such as schizophrenia and are incapable of working are entitled to receive welfare benefits up to 1000 euros per month while they receive their medication for free (Public Relief Scheme). Those welfare recipients are also exempted from various other taxes that non benefits’ recipients pay on a yearly basis. Additionally, mental health patients are entitled to free medical treatment depending on their income and they also have free access to community mental health services regardless of their economic status. As a consequence, a substantial amount of the financial burden passes from the family to the government.

These benefits may also correlate with other aspects of low family burden. In particular, by providing free medical care the government ensures that all patients have an equal access to medical care, which makes possible low tension and supervision for the families with members who suffer from a severe mental illness.

However, the benefits that people with severe mental illness receive may fall an easy prey due to the financial crisis. In particular, the austerity measures that are being discussed the past few months and proposed legislative measures towards this direction are rather pessimistic for the future of the current welfare system. A system where the attention is being on money transfers rather than services. Along these lines is the situation that exists in the field of health which is mainly characterised by a mixed system of public and private health services. However, in Cyprus there is no National Health System. The current policy of the Ministry of Health lies on the principle of mandatory participation, equality, social justice and solidarity that is mainly achieved through the increased state intervention [43].

The above situation represents a major policy issue in the debate on the reform of traditional welfare states and social policies, given that it may affects a broad range of interested target groups (unemployment claimants, long term unemployed, welfare claimants, people excluded due to mental or physical disability). During poor economic times vulnerable social groups are likely to experience higher rates of unemployment and underemployment, as well as bear the brunt of cutbacks in government spending on health and social services [4]. Access to health and other social services is likely to decrease and this will affect the disability, morbidity and mortality rates associated with these groups. When that happen it is expected that family burden will increase significantly especially in cases as Cyprus where the burden will be transferred from the state to the family.

However, government should be aware that by doing so on the one hand they may decrease substantially the welfare of the patients with a severe mental illness, but on the other hand they may increase the burden that their families will have to curry due to the increase of costs in the provision of treatment. As Batic [44], p.158 claims:

“The economic crisis has had a negative effect on mental and physical health, and has brought the danger of deepening health inequalities. The influence of the crisis will vary depending on EU member states’ initial health situations and capacities to deal with the challenges. Increased demand, coupled with large budgetary pressures, make it urgent to increase the efficiency of healthcare systems, along with ensuring access to quality healthcare for all”

It is thus, clear that on the basis of the effects of the global economic crisis on the labor market, social insurance funds and state budgets, EU member states will have to intensify welfare state reforms, which has been a demand for years. Reduced social benefits and increased taxes and contributions are unavoidable if the current state of affairs remains unchanged, especially in the group of high risk countries, which make up almost one half of the EU member states.

## Conclusions

It is becoming obvious from what it has been described on the previous sections and on the basis of the findings of this study that the family burden in Cyprus will increase dramatically if the current state of the mental health system and the overall welfare state will not adopt to the pressures of the economic crisis. As Kleinman [45], 2009 claims the global mental health is a reflection of failure of humanity. He rightly pointed out that families of the individuals with mental illnesses are not only sharing the suffering from the illnesses but are actually key caregivers for the affected individuals. As professional services for helping the families are inadequate [37], families simply fail to offer the desired care by themselves.

Consequently, families under great stress due to the reasons described throughout this paper would give up and reject the mentally ill individuals who would become socially out-casted. The mentally ill individuals would rarely participate in daily social activities such as festivals, marriage, celebrations, or even family shopping. Such a nonperson life ends with the mentally ill individuals becoming further stigmatized. Therefore, mental health systems and consequently professionals need to develop more innovative programs for families. Instead of only supporting the families and easing their burdens through monetary provisions, it could be more effective to involve the families as active members of the health care team by assigning specific tasks for the families and allocating the necessary resources for performing such tasks. As Igberase et al., [46], 2010 have clearly shown that caregivers of patients with schizophrenia experience immense burden. They also suggest that Public health education as well as targeted interventions in the area of employment, financial and other support for persons with mental disorders would help to ameliorate this burden. According to our study findings and to what Igberase et al., (2010) suggest, health systems need to aim to the development of psychosocial provisions for both family caregivers and patients as to decrease the family burden rates and increase the possibility of smooth transition to the society.

## Endnotes

^a^Austerity measures proposed under the Memorandum of the Troika for the RoC.

^b^World Health Organisation (2000) Operational Guidelines for Ethics Committees That Review Biomedical Research, Geneva.

## Competing interests

The authors declare that they have no competing interests.

## Authors’ contributions

MA carried out the collection and analysis of data and CP contributed in drafting the manuscript. AP contributed to the design of the study. All authors read and approved the final manuscript.
